# The Alkaline Treatment of Rheumatism

**Published:** 1859-03

**Authors:** B. F. Schneck

**Affiliations:** Lebanon, Pa.


ARTICLE IV.

### THE ALKALINE TREATMENT OF RHEUMATISM.

BY B. F. SCHNECK, M.D., OF LEBANON, PA.

To Dr. Fuller* belongs the credit of having published, probably, the most rational as well as successful treatment of this very common and painful affection, with which we are acquainted. The great merit of the plan, and which entitles it to a ready acceptance at our hands, lies in the fact that a certain theory which it assumes is proven to be correct—palpably, to the senses, in every case—by the treatment recommended. The object of this paper being only to adduce facts and cases confirmatory of the plan in question, it will not be necessary to go into a detailed statement of the author's views, further than may be necessary to make them intelligible to those who may not have seen his work.

That morbid state of system, which all must have observed in rheumatism, manifested by acid saliva, acid urine, and intensely sour-smelling perspiration, is regarded as the *causus morbi*, and its removal the sure guarantee of a speedy recovery. When treating of the seat of rheumatism, the author thus, in a few words, elaborates his entire theory:

"The reason of this predilection of the rheumatic poison for the fibrous and fibro-serous textures throughout the body, is not at first sight obvious, nor, indeed, after the most careful consideration, can we assign to it other than a conjectural cause. But it is worthy of note, that the textures most commonly implicated in rheumatism are all examples of the albuminous and gelatinous tissues, from the decomposition of which, in the wear and tear of the body, are formed those secondary organic compounds, the lithic and lactic acids, with which gout and rheumatism are intimately connected. And as it is but consistent with our knowledge respecting the processes of nutrition

---

* On Rheumatism, Rheumatic Gout, and Sciatica; their Pathology, Symptoms, and Treatment. By Henry William Fuller, M.D., Cantab., Fellow of the College of Physicians, London; Assistant Physician to St. George's Hospital, etc., etc. London, 1852. 8vo., pp. 403.

and assimilation, to suppose that each tissue selects from the blood and appropriates to itself such matters as correspond with it in chemical constitution, we may readily conceive that some peculiar attraction may be exerted by the fibrous and fibro-serous textures for compounds, such as lithic and lactic acids, to which they bear so strong an affinity."

These acids—the lithic and lactic—and possibly others—to whose presence in the blood, and hence in the secretions, the author modestly and only *conjecturally* ascribes the existence of the disease, are undoubtedly its true cause. In every case which has come under my observation for the last five years, my attention has been kept specially directed to this point; and I have found, invariably, that as long as the acid state of the system was not neutralized, and the various secretions continued to show acid reactions, there was no real, permanent improvement. But no sooner was the blood saturated with the neutral salts, and the various secretions showed alkaline reactions, than recovery might be confidently and speedily predicted.

However successful nitrate of potassa, guaiacum, mercury, colchicum, quinine, lemon juice, and, more lately, veratrum viride, may occasionally be in rheumatism, it will be admitted by all that they frequently fail; and it is claimed for the alkaline treatment, that it, more than any other method, opposes the most rational remedies to the disease, and cures upon intelligent and intelligible principles. Quinine, it is said, acts beneficially in this affection, in that it reduces the frequency and force of the pulse, and so moderates the rheumatic fever; and the same is lately claimed, to a still greater degree, for the energetic veratrum viride, as though to diminish the frequency of the heart's action and lessen its impulse, were to counteract the cause upon which these mere effects depend. Beyond this, and deeply below this superficial view, goes the method of Dr. Fuller, which presupposes, for successful treatment, a vital but not the less a chemical action in the economy—a real neutralization of an acid by an alkali. It is the object of this paper to bespeak for the plan a favorable consideration, and a more general adoption.

As regards the treatment, Dr. F. says: "The form in which

I usually administer the remedies, is that of a simple saline or nitre draught, to which, if the patient be a person of average strength and robustness, bathed in profuse perspiration, with red, swollen, and exquisitely painful joints, a furred tongue, loaded urine, and a full and bounding pulse, I usually add from two to three drachms of the potassio-tartrate of soda, ten or fifteen minims of the vinum colchici, from fifteen to twenty minims of the vinim antimonii, and from ten to fifteen minims of the tinctura opii, or of Battley's sedative solution, to prevent the salt running off by the bowels. This draught is repeated for the first twelve or twenty-four hours at intervals of three or four hours." * * *

After that the intervals are lengthened; and generally after the lapse of three or four days, the amendment is so decided as to justify a resort to quinia, which, either alone or in conjunction with some of the alteratives, is soon followed by complete recovery.

With reference to local remedies, Dr. F. informs us that he usually employs the following: ℞ Potasso carbonat. ℥i.; liquor. opii sedative ʒvi.; aqua rosæ ℥ix. "It is applied by soaking thin flannel in the solution, and applying this to the inflamed parts, and then enveloping the whole in gutta percha."

The several cases which follow have been selected from a large number on my record, in illustration and confirmation of the alkaline treatment, as taught by Dr. Fuller.

*Case I. Acute Articular Rheumatism, with Sequelæ.*—1855. The wife of Jno. B. M—— was attacked with acute dysentery, on Sept. 10th, from which she convalesced, after four weeks' illness, very much reduced. About the 18th of October, she felt stiffness of both knees; soon, also, great swelling, with heat, redness, and acute pain manifested themselves. High fever now appeared, and the case was decided enough. How would the usual remedies for rheumatism, as nitre, colchicum, Scudamore's mixture, etc., answer in this case, where the alimentary canal was still in a very irritable condition from the dysenteric attack? I tried small doses of Dover's powder and calomel for a few days, from apprehensions as to the tolerance of severer remedies by the bowels; but finding not the least improvement, I made the usual and favorite Rochelle salt solution, guarding its purgative tendency by full proportions of laudanum. On the second day the patient felt better. The elbows were now effected; and after a few days the right wrist and ankle, then the right hip (an unusual place), then again the knees, and so on several times in rotation, but every successive attack milder than the former. In two weeks the patient left her bed convalescent, but complaining of slight pain in the right eye. Examining it, I found sclerotites of considerable severity. Knowing it to be rheumatism purely, I resolved to try a novel experiment; and accordingly merely continued the alkaline treatment, with the addition of Dover's p. 10 grs., cal. 1 gr., at bed time. Neither general nor local depletion, nor any topical applications were resorted to. Every day the redness became fainter, the eye felt better, and in eight days it was perfectly well, without even purgatives or blisters.

Upward of a week afterward, I was sent for again, to see a new phase of this strange case. The ball of the great toe of the right foot was affected with acute gout, well developed. After a brisk dose of Scudamore's purgative mixture, I gave the usual alkaline solution, with, however, a double proportion of wine of colchicum. In three days the toe ceased to be painful, red, and swollen, and rheumatic gout of the right eye again appeared; the inflammation being of a much higher grade than before. I repeated the purgative as above, and trusted to Rochelle salt, which was given in as large doses as the bowels would bear. The improvement, which was gradual but sure, was helped by Dover's p. and cal. at night; and in two weeks afterward the eye was again natural, both as to function and appearance. The sclerotic coat alone showed slight evidence of inflammation in a faint dullness, or ashy gray color, as compared with the clear white of the uninflamed eye.

I feel confident, from the success attending the treatment of this case, that rheumatic ophthalmia can, in most instances, be more effectually and speedily cured—and with more comfort to the patient, too—by the simple method above detailed, than by bleeding, cupping, blisters, and other energetic measures, or the always-unpleasant infliction of a salivation.

*Case II. Long-standing Sub-acute Rheumatism, with Complications.*—On Monday, Oct. 15, 1855, I saw David W——, aged 40, for the first time, and obtained the following history: In November last, he was attacked with acute rheumatism, for which he was treated by a neighboring physician by the following mixture, principally: whisky one quart, saltpetre half a pound; to this was added a certain quantity (I could not infer how much) of powdered guaiac. The dose was a good, large draught, three times a day. It is natural to suppose that no stomach would hold out long against such heroic medication; and I was told, that before the first prescription was all taken, vomiting, purging, and great gastric distress for a while existed. A second dose, whisky a pint, saltpetre a quarter of a pound, was, however, ordered, and also taken. Some time in the following January, there was a translation to some internal organ, as I should judge from his rather incoherent account, for which he was bled twice, cupped once, and blistered innumerable times, until the "pain in his side" left him. During the spring and summer, the affection was gradually losing its open, inflammatory character, and assuming the sub-acute form. During this time he was seen by a number of physicians, and prescribed for by all, with but moderate success.

I found him as follows: Greatly emaciated; pulse frequent and irritable; skin hot and feverish; tongue very thickly plastered with a yellowish white crust, which, in three or four places, had scaled off, leaving the surface red, smooth, and divested of the papillary structure; bowels very much bound; sleep restless, and much interrupted by the pain of the extremities. Urine scanty, high colored, and very acid. The knees were very painful and slightly swollen; and the metacarpo-phalangeal articulation of the right hand and the wrist of the left were greatly swollen, red, and very sore. The wrist of the right hand was enormously thickened, but not painful.

Put the patient on the use of the following:

| | | | |
|---|---|---|---|
| ℞ | Rochelle salt, | | ℥i. |
| | Sp. nitric ether, | | ℥ij. |
| | Vin. colchici, | aa, | ℥ss. |
| | Vin. antimon, | | |
| | Tinct. opii, | | ʒij. |
| | Aqua, to fill a six ounce vial. | | M. |

*Sig.* A tablespoonful every two or three hours.

To correct the gastro-intestinal disorder, gave the following:

| | | |
|---|---|---|
| ℞ | Pulv. rad. gentian, | ℥ss. |
| | Pulv. rad. rhei, | ʒi. |
| | Sodæ bi-carbon, | ʒiij. |

M. and divide into four parcels. Half a pint of hot infusion to be made of a parcel; dose, a wineglassful three times a day.

Unguent. tabaci to the painful joints.

*October* 22*d.*—Somewhat better; has less pain; sleeps well; pulse slower and less angry; tongue very thinly coated and clearing; bowels still costive; second and third metacarpo-phalangeal joints of left hand now affected; knees better; feels very hopeful, but is entirely helpless. Continue remedies, with addition of extr. taraxaci.

*October* 30*th.*—Has more pain; not so well in all respects as at last visit. Very costive; increased the quantity of rhei. Continued alkaline solution, with larger proportion of Rochelle salt.

*November* 10*th.*—In statu quo. Increased the strength of alkaline solution, and gave, twice a day, cal. $\frac{3}{4}$ gr., opium $\frac{1}{2}$ gr., ipecac $\frac{1}{4}$ gr. Observing several white stains upon his pants, in such a situation as might be produced by drops of urine, I inquired, and was told that some drops had lately fallen there, and left the spots in question. He had observed, during the summer, that when urinating on the back porch, the place accidentally wetted on the boards, looked, when dry, as though very fine, dry salt had been sprinkled over it. I tested his urine for diabetes mellitus, but found no traces of sugar.

*November* 16*th.*—Gums slightly touched. Discontinue cal. and opium, but continue solution. Alkaline lotion to joints. Ordered Scudamore's mixture for the first time.

*November* 18*th.*—Was sent for in haste; severe purging, with cramp of stomach, caused, perhaps, by Scudamore's mix. Morphia soon quieted the intestinal commotion.

*November* 24*th.*—Symptoms of improvement. Continue solution; tr. iodine to joints, with alkaline and anodyne lotion.

*December* 1*st.*—Much better, as manifested by the softened slow pulse, the freedom from articular pain, and a subsidence of the swelling in all the joints. Is able to walk about the room with a crutch. Syr. iodide iron forty drops, three times a day; bi-chloride mercury one-sixteenth gr. every evening. Ordered a warm bath, impregnated with sulphuret potassa, three times a week, half an hour at a time. Patient lies in it at full length, derives the greatest comfort and ease from it, and would continue it all day long, if permitted.

*December* 21*st*.—Walked to-day without staff or assistance. Joints still somewhat painful, but swelling all gone. Nutrition is very well performed; face is filling up, and color is good. Continue remedies, with occasional dose of alkaline solution.

From this time forward, until the latter part of April, when I made him my last visit, the improvement was slow, but steady, except occasionally a sub-acute swelling of the small joints of the fingers, generally only of short duration. At last visit gave him two grs. iron by hydrogen, three times a day, which, with warm, sulphuretted baths, were continued for some months. He has had no relapses; has been for the past eighteen months, and is now, again actively engaged in various business callings; and his present vigorous health gives no evidence whatever of the unlucky *pickle* in which I originally found him, and which had well nigh effectually *cured*, not only his stomach, but the entire organism.

If there were any use in multiplying cases, I might adduce many other and similar examples, illustrating the favorable results of this treatment.

In sub-acute and chronic rheumatism, its beneficial effects are equally decided. In illustration, I will quote

*Case III.*—I was consulted, several weeks ago, by J. B——, for a very painful and lamed condition of his right shoulder. Had felt it coming on for upward of a week; and it was now so bad that the least motion of the arm was agonizing. There was no swelling nor fever. Gave Rochelle salt ℥ij., Dover's p. grs. viij., three times a day, with 30–40 drops of wine of colchicum to each dose; and applied the alkaline and anodyne lotion locally, covering the part with oiled silk. In two days the pain became quite tolerable, and the man got sound, refreshing sleep at night; and in eight days he had recovered the full use of his limb, driving his own carriage, and experiencing only a feeling of numbness, which in a short time yielded to a strong camphorated liniment.

